# Evaluation of Alabama phosphorus index using edge‐of‐field monitoring data

**DOI:** 10.1002/jeq2.70152

**Published:** 2026-02-13

**Authors:** Anjan Bhatta, Rishi Prasad, Debolina Chakraborty, Dexter B. Watts, Henry A. Torbert, Peter Kleinman

**Affiliations:** ^1^ Department of Crop, Soil, and Environmental Sciences Auburn University Auburn Alabama USA; ^2^ Department of Biosystems Engineering Auburn University Auburn Alabama USA; ^3^ USDA‐ARS National Soil Dynamics Lab Auburn Alabama USA; ^4^ USDA‐ARS‐Soil Management and Sugarbeet Research Unit Fort Collins Colorado USA

## Abstract

Phosphorus index (P‐index) was developed to assess field vulnerability to phosphorus (P) loss and guide P management decisions. The original structure of the P‐index was additive, and with continued refinement, multiplicative and component‐based indices were developed. Alabama adopted the additive version in early 2000; however, the tool was never tested for its performance. The objectives of this study were to (i) evaluate the Alabama P‐index using edge‐of‐field P loss data, (ii) test if multiplicative (Tennessee) and component‐based (Georgia) P‐indices perform better, and (iii) improve and test the performance of a modified Alabama P‐index. We evaluated the performance by examining the strength and directional relationship between P‐index scores and annual P loads. The Alabama P‐index showed weak correlations (*r* < 0.50) between risk scores and measured dissolved reactive phosphorus (DRP), total particulate phosphorus (TPP), and total phosphorus (TP) loads. Additionally, directional inaccuracies were observed, indicating that the index misclassified the relative risk of P loss. Further, we evaluated multiplicative and component‐based indices but found similar discrepancies between predicted risk scores and actual P loading. Subsequently, we modified the Alabama P‐index by replacing soil test P with the phosphorus saturation ratio and substituting the underground outlet system factor with the timing of P application. Minor adjustments to weighting factors were made. The modified P‐index demonstrated statistically significant correlations (*r* > 0.51) and directional alignment with DRP, TPP, and TP loads, suggesting it can serve as a reliable interim tool for assessing P losses. Future research should focus on restructuring and validating a component‐based P‐index tailored to Alabama's agricultural systems.

## Core Ideas

- The Alabama phosphorus index (P‐index) risk category showed poor alignment with measured P loads, indicating directional inaccuracies.
- The modified Alabama P‐index showed directional accuracies and can be used as an interim tool for P loss risk assessment.
- Component P‐index shows promise and warrants further restructuring to suit Alabama's specific conditions.

## Plain Language Summary

Phosphorus loss from agricultural fields contributes to water quality deterioration of freshwater bodies. To address this issue and assess the risk of phosphorus runoff, the phosphorus index was developed as a management tool. In Alabama, the phosphorus index was implemented in the early 2000s; however, it was never evaluated whether the tool accurately captures phosphorus loss risk. The objective of this study was to assess how well the risk categories defined by the Alabama phosphorus index align with actual phosphorus losses observed from agricultural fields. We identified that the Alabama phosphorus index risk rating was poorly aligned with actual edge‐of‐field phosphorus loss, which indicated the need for its refinement. We made the first attempt to modify the parameters in the current phosphorus index and compared it with edge‐of‐field phosphorus losses. The modified version is better aligned with phosphorus loss risk and can be an interim tool for P loss risk assessment in Alabama farms.

**Abbreviations**
DRP: dissolved reactive phosphorus
P‐index: phosphorus index
PSR: phosphorus saturation ratio
STP: soil test phosphorus
TP: total phosphorus
TPP: total particulate phosphorus

## 1 INTRODUCTION

Phosphorus (P) losses from agricultural lands are a leading cause of eutrophication worldwide (Kuma et al., 2022; Ross et al., 2022). Given the growing body of evidence on eutrophication and its socioeconomic consequences, minimizing agricultural P loss has been a critical priority since the 1980s (Dodds et al., 2009; Lucas et al., 2023; Sharpley et al., 2003). Recognizing the need to address P losses, the USDA‐NRCS initiated the development of an indexing procedure that integrated both source and transport factors influencing P movement (Osmond et al., 2023). In response, the concept of a P loss risk assessment tool was introduced during a 1990 meeting by a group later recognized as the phosphorus index (P‐index) Core Team. Their efforts led to the release of the first version of the P‐index in 1993, known as the Lemunyon and Gilbert model. The primary goal of the P‐index was to identify critical source areas within watersheds, enabling targeted mitigation strategies to reduce agricultural P losses through runoff (Lemunyon & Gilbert, 1993).

To address water quality concerns stemming from agricultural P inputs via fertilizers and manure, the USDA Natural Resources Conservation Service (USDA‐NRCS) incorporated guidance within its 590 Nutrient Management Conservation Standard. This directive encouraged states to adopt one of three approaches: (i) a fixed soil test P threshold, (ii) a water quality‐based soil test P threshold, or (iii) the P‐index (Osmond et al., 2023; Sharpley et al., 2003). However, there was no mandate to validate these tools against actual water quality monitoring data. Subsequently, many US states adopted and modified the P‐index, with the last major round of updates occurring in 2010 (Nelson & Shober, 2012; Sharpley et al., 2011).

The first version of the P‐index was additive, where scores of all factors were summed to determine the final score (Osmond et al., 2012). This approach failed to account for interactions between source and transport processes, prompting the development of a multiplicative model by Gburek et al. (2000), which calculated risk as the product of source and transport factor scores. Despite improvements, the multiplicative model's separation of source and transport factors showed inconsistencies when compared to process‐based P loss models (Bolster et al., 2012). To better reflect P loss dynamics, a component‐based approach was later introduced. This method evaluates particulate and dissolved P transport separately and calculates P loss quantitatively for each component. Several states, including North Carolina, Iowa, Georgia, Missouri, and Wisconsin, have adopted this approach for more nuanced assessments (Bolster et al., 2012).

Variations in P‐index formulations and the flexibility granted to individual states for customization led to significant inconsistencies in P loss ratings and associated management recommendations across the U.S. (Osmond et al., 2023; Sharpley et al., 2012). Long‐term evaluations indicated that P‐index‐based management strategies did not consistently result in measurable improvements in water quality, raising concerns about their overall effectiveness (Nelson & Shober, 2012; Sharpley et al., 2017). These findings prompted the USDA‐NRCS to reconsider its reliance on P‐indices, exploring simulation models such as APEX, APLE, and TBET as alternatives. However, Sharpley et al. (2012) emphasized the importance of critically assessing existing P‐indices before replacing them. This led to a nationwide evaluation effort funded by NRCS to systematically assess the performance and reliability of current P‐index tools (Sharpley et al., 2017). Results from this effort showed that multiplicative and component‐based indices performed better or similar to the process‐based models, while additive indices were less robust (Osmond et al., 2017). Consequently, USDA‐NRCS mandated states to evaluate, revise, and restructure their P‐indices under the revised Nutrient Management Practice Standard (Practice Standard 590; USDA‐NRCS, 2011).

Evaluation methods for P‐indices include comparisons with other indices, sensitivity analyses, plot‐scale simulated rainfall studies, edge‐of‐field monitoring, and watershed‐scale assessments (Nelson & Shober, 2012). Some studies also utilize numerical modeling and simulated datasets (Bolster et al., 2014; Fiorellino et al., 2017; Osmond et al., 2017; Vadas et al., 2012). However, long‐term field‐scale data are considered the most reliable for evaluating P‐index performance (Sharpley et al., 2012).

The Alabama P‐index, implemented in the early 2000s, is qualitative and categorizes relative P loss risk for nutrient management. The additive structure was selected for its simplicity and ease of use. Despite its long‐standing application, the Alabama P‐index has not been comprehensively evaluated for accuracy or reliability. Notably, Osmond et al. (2006) identified substantial differences in P loss risk ratings across southern states, with Alabama's Index generally assigning higher risk scores. Subsequent evaluations confirmed these discrepancies. In a multi‐state comparison, Osmond et al. (2012) reported that component‐based (Georgia, North Carolina) and multiplicative (Arkansas, Florida, and South Carolina) indices showed moderate to strong correlations with P loads (*r* ^2^ = 0.50–0.97), while the Alabama P‐index lacked predictive strength and exhibited a negative directional relationship with dissolved P losses. These findings prompted the need to evaluate, restructure, and revise the Alabama P‐index.

This study utilized edge‐of‐field monitoring data from three sites in Alabama with six watersheds (size ranging from 1.87 to 37.23 ha) to evaluate and refine the state's P‐index, ensuring the risk rating for fields using P‐index aligns with the measured P loadings. The specific objectives of this study were to (i) compare Alabama P‐index scores with measured annual loads of dissolved reactive phosphorus (DRP), total particulate phosphorus (TPP), and total phosphorus (TP) in runoff to assess alignment with actual P losses; (ii) evaluate the suitability of the multiplicative and component‐based P‐indices for Alabama farms; and (iii) propose an interim modified P‐index to improve P loss risk assessment. Additionally, this study outlines future research directions for refining the index structure to develop a robust and quantitative P assessment tool tailored to Alabama's agricultural systems.

**Core Ideas**

- The Alabama phosphorus index (P‐index) risk category showed poor alignment with measured P loads, indicating directional inaccuracies.
- The modified Alabama P‐index showed directional accuracies and can be used as an interim tool for P loss risk assessment.
- Component P‐index shows promise and warrants further restructuring to suit Alabama's specific conditions.

## 2 MATERIALS AND METHODS

### 2.1 Edge‐of‐field monitoring sites

**FIGURE 1:**
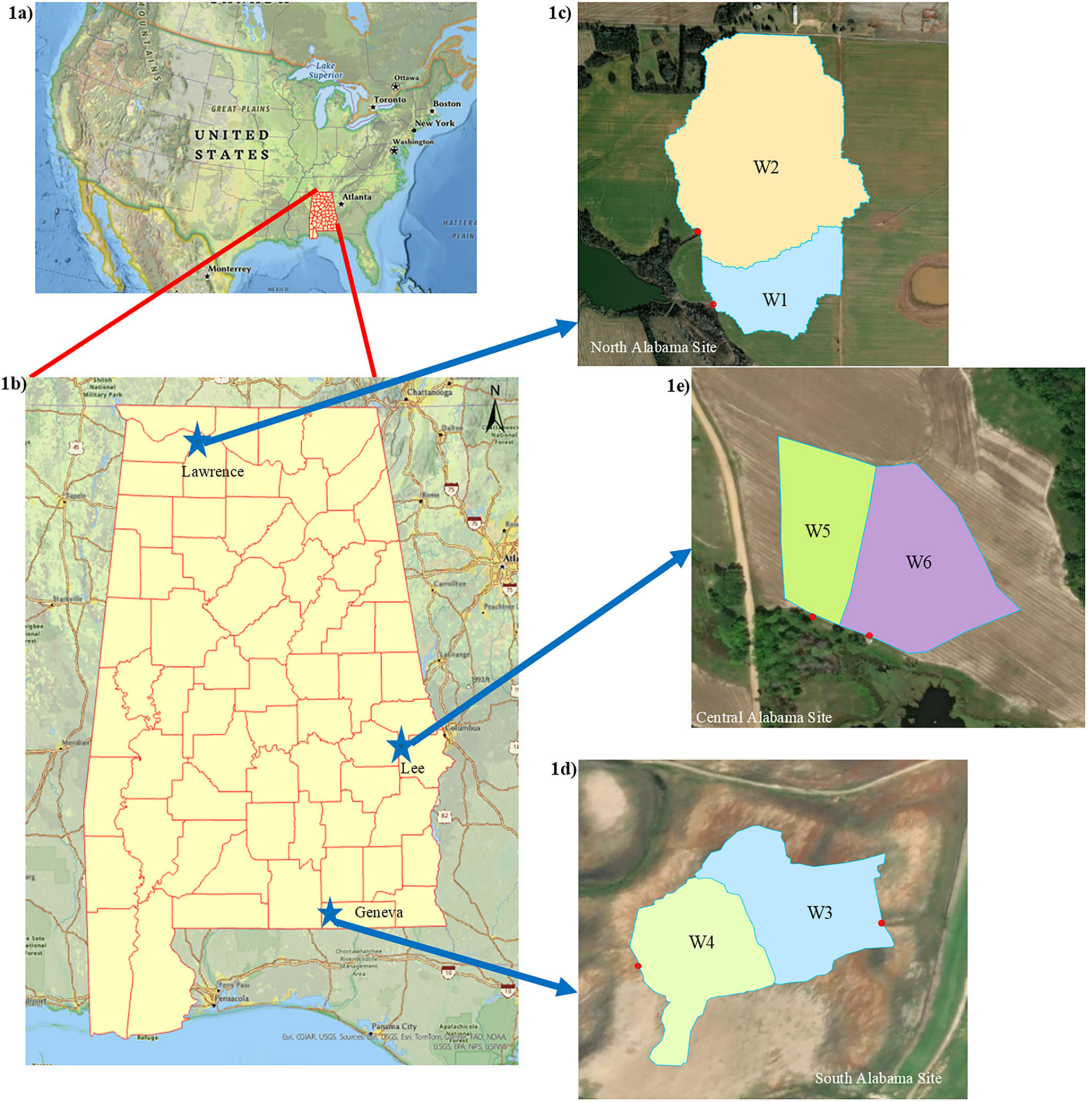
Geographic location of (1a) Alabama relative to the United States; (1b) geographic location of the watersheds within the state of Alabama; (1c) North Alabama sites, Watershed‐1 (W1) and Watershed‐2 (W2); (1d) South Alabama sites, Watershed‐3 (W3) and Watershed‐4 (W4); and (1e) Central Alabama sites, Watershed‐5 (W5) and Watershed‐6 (W6).

Three farms located in North Alabama (Lawrence County), South Alabama (Geneva County), and Central Alabama (Lee County) representing diverse cropping systems and soil types were selected for this study. The North Alabama site is located in Limestone Valley physiographic region, while South and Central Alabama sites are located in the Coastal Plain region. At each farm, two adjacent watersheds were delineated to capture edge‐of‐field P losses. Watershed site locations and boundaries are provided in Figure 1: Watershed‐1 (W1) and Watershed‐2 (W2) from North Alabama, Watershed‐3 (W3) and Watershed‐4 (W4) from South Alabama, and Watershed‐5 (W5) and Watershed‐6 (W6) from Central Alabama. The detailed information on watershed size, dominant soil types, cropping systems, and P management is provided in Table S1. Information on field management practices was determined annually through farmer interviews.

Data collection for this study was conducted from April 2021 to March 2024 at Watersheds 3–6, and from May 2021 to May 2024 at Watersheds 1 and 2, based on site‐specific cropping patterns. The 30‐year average annual precipitation data was retrieved from the nearest weather stations. Based on 30‐year historic data, the North Alabama (W1 and W2) received 1373 mm (recorded at Muscle Shoals AP), South Alabama (W3 and W4) received 1572 mm (recorded at Westville, WWH20), and Central Alabama (W5 and W6) received 1436 mm (recorded at Lafayette 2 W). The onsite annual precipitation recorded at North, South, and Central sites ranged from 853 to 1390 mm, 919 to 1315 mm, and 1145 to 1195 mm, respectively.

### 2.2 Runoff monitoring, sample collection, and analysis

Edge‐of‐field monitoring stations were installed at the outlet of each watershed to measure runoff and collect water samples. H‐flumes were installed at Watersheds 3–6, while plastic culverts were used at Watersheds 1 and 2 to accommodate larger drainage areas and higher runoff volumes. The H‐flumes at Watersheds 3, 4, and 6 were 0.91 m in height, with a maximum discharge capacity of 0.87 cubic meters per second. At W5, a smaller H‐flume (0.76 m in height) was installed, with a discharge capacity of 0.55 cubic meters per second. For Watersheds 1 and 2, plastic culverts with a diameter of 0.53 m were used. Discharge from the H‐flumes was monitored using 730 Bubbler Modules (ISCO, Teledyne), while discharge from the culverts was measured using 750 Area Velocity Flow Module Sensors (ISCO, Teledyne). Runoff water samples were collected using ISCO 6712 autosamplers (Teledyne ISCO Inc.), which were programmed to initiate sampling once runoff depth reached a threshold of 0.05 m. After collection, the autosampler's built‐in refrigeration system maintained the samples at 4°C to preserve water quality for subsequent analysis. The autosamplers were programmed to initiate runoff sampling once a predetermined threshold of 0.05 m in flow depth was reached. This threshold was selected to ensure the capture of both small and large runoff events. For each precipitation event, runoff water was sampled using a time‐proportional sampling method. Four aliquots were pumped into one of the 14, 900 mL collection bottles, representing a single composite sample. Once a bottle was filled with four aliquots, the autosampler automatically switched to the next bottle. Collected samples were retrieved within 24 h and transported to the laboratory in ice‐packed containers to preserve sample integrity. In the laboratory, a subset of each sample was used for phosphorus analysis. Water samples were filtered through a 0.45 µm membrane and analyzed for DRP within 48 h of collection. TP was determined by digesting unfiltered samples using the acid ammonium persulfate method (EPA Method 365.4; USEPA, 1983). Dissolved total phosphorus (DTP) was measured by digesting filtered samples using the same protocol as TP. All phosphorus analyses were performed using the ammonium molybdate‐ascorbic acid method (EPA Method 365.1) with a flow injection analyzer, following the procedure outlined by Murphy and Riley (1962). TPP was calculated as the difference between TP and DTP (Sobota et al., 2011).

Runoff data for W5 in 2021 were unavailable due to instrument malfunction and were therefore excluded from the analysis. Overall, the dataset used in this study represents approximately 45%–60% of the total runoff events across the monitored watersheds. Several low‐intensity rainfall events did not generate sufficient runoff to trigger the autosamplers, resulting in missed sample collection. Additionally, a few high‐flow events were not sampled due to equipment malfunction or temporary clogging of sampling tubes. Despite these limitations, the dataset effectively captures the dominant P export dynamics through edge‐of‐field monitoring. Potential biases arising from unrepresented events are acknowledged and discussed in the Results and Discussion section, where their implications for broader interpretation of the findings are contextualized and addressed.

### 2.3 Phosphorus load calculations

The concentration of DRP, TPP, and TP in runoff water was expressed as an event mean concentration (mg L^−1^) for individual events and calculated using Equation (1). The total amount of P loss in an individual rainfall runoff event was expressed as load (kg ha^−1^) and calculated using Equation (2). The annual loads were calculated as the sum of all individual events occurring within respective growing season each year.

(1) $$\mathrm{Event}\;\mathrm{mean}\;\mathrm{concentration}\;\left(\mathrm{mg}\;L^{-1}\right)=\frac{\sum_{i=1}^{n}\mathrm{Vi}\;\times \;\mathrm{Ci}}{\sum_{i=1}^{n}\mathrm{Vi}}$$

where Vi is discharge volume corresponding to sample *i* and Ci is concentration in sample *i*.

(2) $$\begin{array}{ccc} \mathrm{Load}\;\left(\mathrm{kg}\;ha^{-1}\right) & = & \mathrm{Event}\;\mathrm{mean}\;\mathrm{concentration}\\ & & \times \;\mathrm{total}\;\mathrm{discharge} \end{array}$$

### 2.4 Soil sampling

Soil samples from the watersheds were collected every year. Soil sampling was carried out on 0.8 ha grids for W1 and W2 and 0.4 ha grids for other watersheds. The soil samples were collected at four locations within each grid and composited to represent individual grids. The samples were collected at 0‐ to 15‐cm depth. The grid map for the soil sample collections is presented in Figure S1. The soil samples were collected after the termination of cover crops (South and Central Alabama sites) and the harvest of winter wheat (North Alabama site) in the year 2021. In the following years, 2022 and 2023, soil samples were collected after the harvest of cash crops in October/November. The soil samples were air dried, ground, passed through a 2 mm screen, and extracted for Mehlich‐1 P (Mehlich, 1953) and Mehlich‐3 P (Mehlich, 1984), which were performed using standard protocol as stated in Bhatta et al. (2021) and analyzed using inductively coupled argon plasma spectroscopy (ICAP, Spectro Ciros, Spectro Analytical Instruments).

### 2.5 Additive structure for Alabama P‐index

The Alabama P‐index is structurally similar to the Lemunyon and Gilbert (1993) model with few modifications in the original source and transport factors (USDA‐NRCS, 2014). The current version of the Alabama P‐index is presented in Table 1. It includes four source factors (soil test P, P application rate, method of application, and grazing animals) and six transport factors (underground outlet system, erosion rate, hydrologic soil group, field slope, P application distance to water, and vegetative buffer width), plus a receiving category for proximity to 303d‐listed watersheds. Each of the 11 factors is assigned a weighting factor ranging between 1 and 3 and discrete value ratings (0, 1, 2, 4, and 8) based on field characteristics. The weighting and value ratings are multiplied for each factor and are summed up to get the final P‐index score. Finally, the P‐index scores are assigned into one of the five ratings: low (≤65), moderate (66–75), moderately high (76–85), high (86–95), and very high (≥96), and P loss risk is interpreted, which further guides P application rate.

**TABLE 1:** Alabama phosphorus index.

		Value rating					
		Low					Very high
Source characteristics		Weight	0 Point	1 Point	2 Points	4 Points	8 Points
1. Soil test value		1		<0.05	0.05–0.09	0.1–0.15	>0.15
2. P application rate (lbs/P_2_O_5_/ac/year)	Traditional application	3	**…**.	<60	60–120	121–180	>180
	Precision application	2	**…**.	<60	60–120	121–180	>180
3. Phosphorus application method		3	….	Injected deeper than 2 "	Incorporated within 3 days, sprinkler applied or surface applied treated	Surface applied and incorporated within 4–30 days	Surface applied, not Incorporated
4. Grazing animals		1	None	No access to water and not fed in sensitive area	Restricted access to water and not fed in sensitive area	Unlimited access to water and/or fed in sensitive area <100 animals	Unlimited access to water and/or fed in sensitive area >100 animals
**Transport characteristic**							
5. Underground outlet system		3	None	Runoff passes through a grass filter strip before leaving the system	Outlet empty into grass waterways	<30% of field has outlets emptying into drainageways or water bodies	>30% of field has outlets emptying into drainageways or water bodies
6. Erosion rate (t/ac/year)		3	<3	2–5	5–10	10–15	>15
7. Hydrologic soil group	Common soil health	3	**…**.	A	B	C	D
	Improved soil health	2	**…**.	A	B	C	D
8. Field slope (%)		1	<1%	1%–3%	3%–5%	5%–8%	>8%
9. P application distance to water (ft)		3	>400	201–400	101–200	50–100	<50
10. Vegetative buffer width (ft)		2	≥50 or not required	30–49	20–29	10–19 t	<10
**Receiving water categories**							
11. Impaired outstanding or critical habitat waters (ft)		3	Not in watershed	>400	201–400	101–200	<100

Risk categories for phosphorus loss		
Total points from P index	Phosphorus application rate	Generalized interpretation of P index
≤65	Nitrogen rate	**LOW** potential for P movement from the field. There is a low probability of an adverse impact to water bodies.
66–75	3 × P removal by crops	**Moderate** potential for P movement from the field. The chance of organic material or nutrients getting into water bodies exists. Buffers, setbacks, lower manure rates, cover crops, and crop residue practices alone or in combination may reduce impact.
76–85	2 × P removal by crops	**Moderately High** potential for P movement from the field. The chance of organic material or nutrients getting to water bodies is possible. Buffers, setbacks, lower manure rates, cover crops, crop residues, etc., in combination may reduce impact.
86–95	P removal by crops	**HIGH** potential for P movement from the field and an impact on water bodies.
≥96	No P application	**Very HIGH** potential for P movement from the field and an adverse impact on water bodies.

### 2.6 Comparing different structured P‐indices

For a structured comparison, we selected Georgia's component P‐index and Tennessee's multiplicative P‐index, as both states are contiguous to Alabama and share watershed systems (example: Tennessee River basin, Mobile River basin, Apalachicola‐Chattahoochee‐Flint River basin) and need coordinated nutrient management efforts and relevant cross‐boundary P loss risk evaluations. The P‐index scores for Alabama (USDA‐NRCS, 2014), Tennessee (Walker & Hawkins, 2016), and Georgia (Cabrera et al., 2012) were calculated based on the P‐index guidelines from respective states. The field characteristics for six watersheds that were used to calculate P‐index scores for the years 2021, 2022, and 2023 are provided in Table 2. The data from grid‐based soil samples were used for plotting spatial maps for P distributions using the Kriging tool in ArcGIS Pro version 3.4.2, and the spatial distribution of soil P was classified into distinct categories based on soil test P ratings developed for Alabama soils (Mitchell & Huluka, 2012). The dominant soil test P rating from the spatial maps was used in P‐index calculation (Figure S2). The hydrologic soil group and field slope information were obtained from the NRCS Gridded Soil Survey Geographic (gSSURGO) database. The soil erosion rate was estimated using the RUSLE2 soil erosion prediction (2.7.1.13) software. Phosphorus application distance to water was determined by using the “measure” tool in the USGS StreamStats application (https://streamstats.usgs.gov/ss/), where the closest distance between the edge of the field and the closest water body was determined.

**TABLE 2:** Field characteristics for watersheds (W1–W6) for the years 2021, 2022, and 2023 used to calculate the phosphorus index.

S.N.	Characteristics	North Alabama		South Alabama		Central Alabama	
		2021					
		W1	W2	W3	W4	W5	W6
1	Soil test P (0–6 in.)	Very high	Very high	High	Very high	High	Medium
2	PSR_M3_ (0–6 in.)	0.05–< 0.1	0.05–< 0.1	0.05–< 0.1	0.1–< 0.15	0.05–< 0.1	0.05–< 0.1
3	P application rate (lbs/P_2_O_5_/ac/year)	120	120	125	125	No application	No application
4	P application method	Surface application	Surface application	Surface application	Surface application	Surface application	Surface application
5	P application time	May	May	May	May	None	None
6	Grazing animals	None	None	None	None	None	None
7	Underground outlet system	No	No	No	No	No	No
8	Erosion rate (t/ac/year)	10.72	11.94	9.61	8.12	6	6
9	Hydrologic soil group	B	B	C	C	B	B
10	Field slope	5%–10%	3%–6%	1%–3%	1%–3%	1%–3%	1%–3%
11	P application distance to water (ft)	>400	>400	200	200	>400	>400
12	Vegetative buffer width (ft)	>50	>50	>50	>50	>50	>50
13	Critical habitat waters	Not in watershed	Not in watershed	Not in watershed	Not in watershed	Not in watershed	Not in watershed

2022							
S.N.		W1	W2	W3	W4	W5	W6
1	Soil test P (0–6 in.)	High	Very high	High	Very high	High	High
2	PSR_M3_ (0–6 in.)	<0.05	0.05–< 0.1	0.1–< 0.15	>0.15	0.1–< 0.15	0.1–< 0.15
3	P application rate (lbs/P_2_O_5_/ac/year)	240	240	180	180	90	90
4	P application method	Surface application	Broadcast	Surface application	Surface application	Surface application	Surface application
5	P application time	November/May	November/May	March	March	May	May
6	Grazing animals	None	None	None	None	None	None
7	Underground outlet system	No	No	No	No	No	No
8	Erosion rate (t/ac/year)	2.54	3.29	8.73	8.86	8.5	8.5
9	Hydrologic soil group	B	B	C	C	B	B
10	Field slope	5%–10%	3%–6%	1%–3%	1%–3%	1%–3%	1%–3%
11	P application distance to water (ft)	>400	>400	200	200	>400	>400
12	Vegetative buffer width (ft)	>50	>50	>50	>50	>50	>50
13	Critical habitat waters	Not in watershed	Not in watershed	Not in watershed	Not in watershed	Not in watershed	Not in watershed

2023							
S.N.		W1	W2	W3	W4	W5	W6
1	Soil test P (0–6 inch)	Very high	Very high	High	Very high	Medium	Medium
2	PSR_M3_ (0–6 inch)	0.05–< 0.1	0.05–< 0.1	0.1–< 0.15	> 0.15	0.1–< 0.15	0.1–< 0.15
3	P application rate (lbs/P_2_O_5_/ac/year)	120	120	90	90	120	120
4	P application method	Surface application	Surface application	Surface application	Surface application	Surface application	Surface application
5	P application time	November/May	November/May	May	May	May	May
6	Grazing animals	None	None	None	None	None	None
7	Underground outlet system	No	No	No	No	No	No
8	Erosion rate (t/ac/year)	6.49	5.1	10.44	8.63	5	5
9	Hydrologic soil group	B	B	C	C	B	B
10	Field slope	5%–10%	3%–6%	1%–3%	1‐3%	1%–3%	1%–3%
11	P application distance to water (ft)	>400	>400	200	200	>400	>400
12	Vegetative buffer width (ft)	>50 ft	>50	>50	>50	>50	>50
13	Critical habitat waters	Not in watershed	Not in watershed	Not in watershed	Not in watershed	Not in watershed	Not in watershed

Abbreviation: PSR, phosphorus saturation ratio.

### 2.7 Modifications to Alabama P‐index

As a starting point, we utilized the existing Alabama P‐index, maintaining its additive structure, original P loss risk factors, value ratings, and the overall sum of weighting factors. We then introduced targeted modifications by adjusting selected parameters and their associated weightings. The most significant change involved replacing the soil test phosphorus (STP) metric with the phosphorus saturation ratio (PSR), which provides a more accurate representation of a soil's potential contribution to P loss. Recent research emphasizes the importance of incorporating phosphorus sorption characteristics into environmental P loss risk assessment tools (Kleinman, 2017; McDowell & Haygarth, 2024). In particular, the P saturation ratio better reflects the dissolved P concentration in runoff and has been identified as a more effective tool than soil test P for environmental P loss risk assessment (Nair, 2014). Therefore, to better capture P loss risk, we replaced soil test P with P saturation ratio in the Alabama P‐index. The P saturation ratio was calculated using Mehlich‐3 extractable P, Fe, and Al concentration, following the equation of Chakraborty et al. (2021). The spatial distribution of the P saturation ratio was used to represent the ratings for the P saturation ratio in the watershed (Figure S3).

The current Alabama P‐index does not account for the timing of manure applications, a factor known to influence phosphorus loss risk. Given that underground outlet systems are uncommon in Alabama, this factor was removed and replaced with manure application timing. Seasonal risk ratings for application timing were developed using a combination of published literature, methodologies from other state P‐indices, and edge‐of‐field monitoring data specific to Alabama.

Additionally, weighting factors, which significantly influence the sensitivity of individual parameters and overall index performance, were revised to better reflect the relative contributions of key variables. Specifically, the weighting factor for the PSR was set to 3; traditional P application rate to 2; precision application rate to 1; manure application timing to 2; field slope to 2; and distance from P application to water bodies to 2. These adjustments were incorporated into the modified index to calculate updated P loss risk scores.

### 2.8 Data analysis

A total of 17 site‐years of data on annual load of DRP, TPP, and TP were used in the analysis. To evaluate the directional accuracy of the P‐index, that is, how well the index scores align with actual phosphorus losses, we plotted P‐index scores against measured annual runoff loads of DRP, TPP, and TP. This approach allowed us to assess the consistency between predicted risk categories and observed field‐scale phosphorus export. The significance of the relationship between P‐index scores and actual P loads was assessed using the PROC CORR procedure in SAS at *p* = 0.5 The graphs were created using MS Excel 365.

## 3 RESULTS AND DISCUSSION

### 3.1 Annual phosphorus loads among watersheds

**FIGURE 2:**
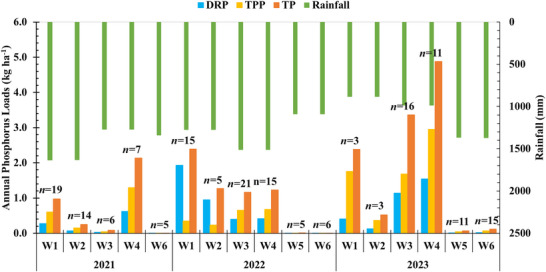
Annual dissolved reactive phosphorus (DRP), total particulate phosphorus (TPP), and total phosphorus (TP) loads from six watersheds for the years 2021, 2022, and 2023. The “*n*” above the bars represents the total number of runoff events captured during the study period.

Annual DRP, TPP, and TP loads from six watersheds (from 2021 to 2023) are presented in Figure 2. The annual DRP loads in surface runoff were highly variable, ranging from <0.01 to 1.93 kg ha^−1^ year^−1^. Similarly, the TPP loads ranged between <0.01 and 2.96 kg ha^−1^ year^−1^, while TP loads ranged from <0.01 to 4.88 kg ha^−1^ year^−1^. The DRP loads constituted 15%–81% of the annual TP loads, whereas TPP accounted for 15%–74% of the annual TP loads. These findings are consistent with previous research by Williams et al. (2016), who reported that surface runoff from 40 agricultural fields yielded DRP loads ranging from 0.00 to 2.78 kg ha^−^^1^ year^−^^1^ and TP loads ranging from 0.00 to 4.12 kg ha^−^^1^ year^−^^1^. However, their cumulative loads (tile and surface runoff) were higher, ranging from 0.03 to 6.88 kg ha^−1^ year^−1^ with tile drainage contributing significantly to TP export. This highlights the influence of soil type and drainage on P losses. Besides, the variability in P loads between sites is greatly influenced by site‐specific source factors such as STP, rate of manure and fertilizer application, and timing of application, as well as transport characteristics including rainfall pattern and runoff, slopes, and vegetation cover (Sharpley et al., 2011). The annual difference in P loads within the same watershed is influenced by differences in cropping system and management practices and rainfall runoff patterns, as observed by Good et al. (2012). For example, in W1, the DRP was the dominant form of P loss in the year 2022, while TPP was predominant in the year 2023, reflecting the effect of poultry litter application rates (9 Mg ha^−1^ in 2022 vs. 4.5 Mg ha^−1^ in 2023). Although annual precipitation was consistently high across all three sites (exceeding 900 mm annually), P loading varied significantly. Notably, Central Alabama sites (Watersheds 5 and 6) exhibited consistently low P losses regardless of year, rainfall amount, or management practices. This was primarily attributed to the loamy sand soil texture, which reduced surface runoff and associated P transport. In contrast, isolated extreme rainfall events at other sites contributed disproportionately to annual P loads. For example, in 2023, W1 experienced only three runoff events, yet recorded higher cumulative P losses compared to W3, which had 16 events. This highlights the importance of rainfall intensity over frequency in influencing P exports. Overall, these findings indicate the critical role of site‐specific factors such as soil type, hydrology, and rainfall patterns in shaping P loss dynamics across agricultural landscapes.

### 3.2 Evaluating the accuracy of Alabama P‐index using edge‐of‐field P loads

**FIGURE 3:**
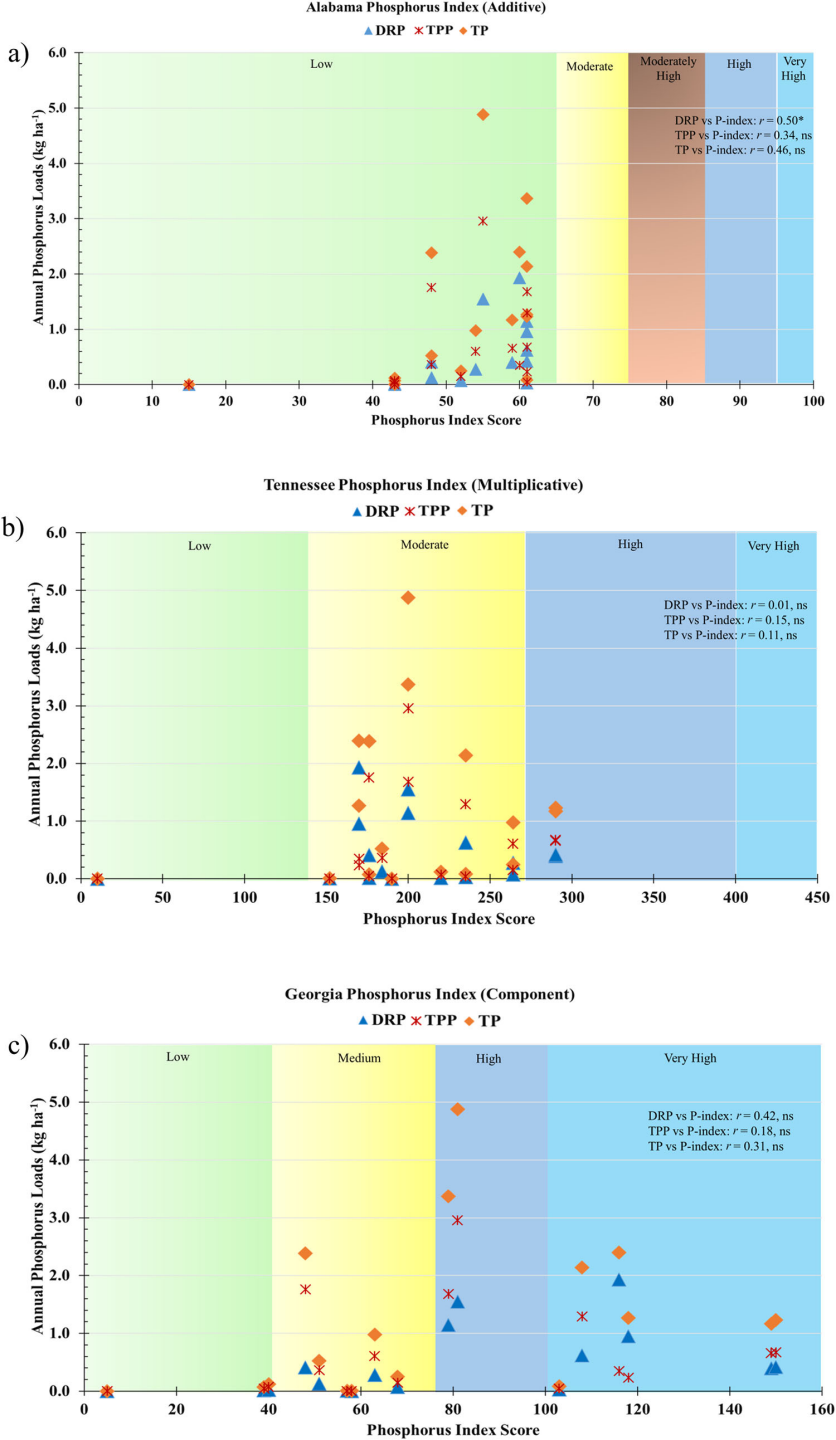
Relationship between annual phosphorus loads (dissolved reactive phosphorus [DRP], total particulate phosphorus [TPP], and total phosphorus [TP]) with phosphorus index score computed using additive, multiplicative, and component‐based phosphorus index (P‐index). (a) Alabama P‐index is additive, where P‐index score <65 is low risk, 66–75 is moderate risk, 75–85 is moderately high risk, 86–95 is high risk, and >95 is very high‐risk categories as differentiated by different colors; (b) Tennessee P‐index is multiplicative, where P‐index score <140 is low, 140–270 is moderate, 271–400 is high, and  >400 is very high‐risk category as differentiated by different colors; and (c) for Georgia, the P‐index is component‐based, where P‐index score <40 is low, 40–75 is medium, 76–100 is high, and >100 is very high‐risk category as differentiated by different colors; ns represent non‐significant (*p *> 0.05) and * represents statistically significant relationship (*p *< 0.05).

Table 3 presents the annually calculated Alabama P‐index scores and corresponding risk ratings for each watershed, alongside the observed ranges of DRP, TPP, and TP loads from 17 site‐years of edge‐of‐field monitoring data. Despite the high variability in the observed annual P loads, the P‐index score for all 17 site‐years was categorized under low risk. Sites that recorded 1.93 kg ha^−1^ year^−1^ of DRP, 2.95 kg ha^−1^ year^−1^ of TPP, and 4.88 kg ha^−1^ year^−1^ of TP loads were categorized as “low risk,” as well as the sites with <0.1 kg ha^−1^ year^−1^ of TP. The relationship between the observed DRP, TPP, and TP loads with the Alabama P‐index score (additive) is presented in Figure 3a. The relationships between Alabama P‐index scores and measured annual loads of DRP, TPP, and TP were not statistically significant (*p *> 0.05), with correlation coefficients (*r*) below 0.50. Since the Alabama P‐index provides relative risk ratings rather than quantitative predictions of P loss, it is essential that its directional accuracy is reliable, that is, higher index scores should correspond to greater observed P losses, and lower scores to lesser losses. However, this expected directional relationship was not observed. Sites with high P loads were often categorized under low risk, indicating a mismatch between P‐index scores and actual field P losses. These findings point to directional inaccuracy in the current Alabama P‐index. Similar results were reported by Osmond et al. (2012), who found that the Alabama index failed to predict dissolved and TP losses in runoff, with r values <0.30. Figure S4 provides site‐specific comparisons illustrating how Alabama P‐index scores align with measured annual P loads across individual watersheds. The current index assigns the highest weighting value (3) to three transport‐related factors: proximity to critical habitat waters, distance from P application to water bodies, and the presence of underground outlet systems. At the North Alabama site (Watersheds 1 and 2), these factors scored zero, resulting in low‐risk index ratings. However, TP loads at these sites varied considerably from <0.25 kg ha^−^^1^ year^−^^1^ in some years to as high as 2.4 kg ha^−^^1^ year^−^^1^ in others, creating ambiguity in risk interpretation. Here, source‐related factors such as application rate and method were the primary drivers of the P‐index score.

**TABLE 3:** The risk category is based on the Alabama phosphorus index and range of dissolved reactive phosphorus (DRP), total particulate phosphorus (TPP), and total phosphorus (TP) loadings associated with the risk categories for all site years of data.

Risk category	Criteria (P‐index score)	Phosphorus loading range (kg ha^−1^)			Number of site years
		Dissolved reactive P	Total particulate P	Total P	
Low	≤65	<0.01–1.93	<0.01–2.96	<0.01–4.88	17
Moderate	66–75	0.39–0.42	0.66–0.68	1.16–1.23	0
Moderately high	76–85	–	–	–	–
High	86–95	–	–	–	–
Very high	≥96	–	–	–	–

At the South Alabama site (Watersheds 3 and 4), all site‐years were classified as low risk despite TP loads exceeding 2.5 kg ha^−^^1^ year^−^^1^. This inconsistency highlights a misalignment between actual P losses and assigned risk categories. In Central Alabama (Watersheds 5 and 6), all site‐years were classified as low risk, which was consistent with observed low TP loads (<0.15 kg ha^−^^1^ year^−^^1^). Similar to the North and South site, source factors dominated the P‐index scores, while transport factor contributions were minimal. These discrepancies between measured P loads and index scores affirm limitations in the additive structure of the current Alabama P‐index. Incorporating interactions between source and transport factors as done in multiplicative and component‐based models may improve the accuracy and reliability of P loss risk assessments (Bolster et al., 2012; Gburek et al., 2000).

The exclusion of unmonitored runoff events may result in a conservative estimate of cumulative annual P loads, potentially influencing the interpretation of results. However, the Alabama P‐index scores and associated risk categorizations for individual sites remain unaffected, as they are derived from site‐specific characteristics rather than measured P loads. Consequently, actual cumulative P losses are likely higher than the values presented. This suggests that additional P loadings from unmonitored events could influence the magnitude of statistical correlations (e.g., may increase or decrease correlation coefficients slightly and significant levels) but will not alter the P‐index risk category or its directional accuracy. Therefore, despite the conservative bias in load estimates, the overall validity of the Alabama P‐index assessment remains robust.

Further, this study examined inconsistencies between Alabama P‐index risk ratings and USDA‐NRCS standardized metrics for P management recommendations (Table S2; USDA‐NRCS, 2011). Based on TP loads, 13 site‐years were classified as low risk (<2.2 kg ha^−^^1^ year^−^^1^), while 4 site‐years fell into the medium‐risk category (2.2–5.6 kg ha^−^^1^ year^−^^1^). These classifications do not align with USDA‐NRCS national guidelines, raising concerns about the consistency and applicability of standardized thresholds. However, national recommendations may be less stringent than some state‐level criteria. For example, Williams et al. (2016) reported that Ohio fields with TP loads below 1.8 kg ha^−^^1^ year^−^^1^ were categorized as medium risk by the state, whereas they would be considered low risk under USDA‐NRCS standards. This discrepancy highlights the potential risk of excessive P application when relying solely on national thresholds, particularly in regions with sensitive water bodies such as Ohio. Stricter regulations may be necessary to safeguard water quality, as demonstrated by the loading thresholds outlined in Annex 4 of the 2012 Great Lakes Water Quality Agreement Amendment for Lake Erie, which recommends limits of <0.29 kg ha^−^^1^ year^−^^1^ for DRP and <1.24 kg ha^−^^1^ year^−^^1^ for TP. These findings highlight the importance of carefully selecting threshold criteria that balance agronomic needs with environmental protection, especially in vulnerable watersheds.

### 3.3 Comparison of edge‐of‐field monitoring data with multiplicative and component‐based P‐index

We selected adjacent state P‐indices to compare the different P‐index structures with the Alabama P‐index using the water quality data. The multiplicative P (Tennessee) and a component‐based (Georgia) P‐indices were selected. The relationships between the multiplicative and component P‐indices and DRP, TPP, and TP loads are presented in Figure 3b,c, respectively. The multiplicative P‐index did not show improved relationships with annual DRP, TPP, and TP loads. The P loss risk category for the studied watersheds shifted to moderate risk. However, the discrepancy remained, as watersheds with <0.01 kg ha^−^^1^ year^−1^ of TP load and those with 4.88 kg ha^−^^1^ year^−1^ of TP loads received the same risk rating. A study by Osmond et al. (2012) found that Tennessee P‐index did not show better correlation (*r* < 0.36) with dissolved P and TP loads in runoff. The driving factors for P‐index scores in the multiplicative P‐index (Tennessee) include erosion rate, P application rate, and application method. The difference in rating categories between additive and multiplicative indices resulted in a shift to medium risk in the multiplicative index. For instance, an erosion rate of 10–15 tons/acre/year is assigned eight points in the Tennessee P‐index, while it receives only four points in the Alabama P‐index. Additionally, the Tennessee P‐index includes application timing, which is not considered in Alabama's index. This discrepancy in the use of source and transport factors between states' P‐indices and the ratings for individual factors can lead to different P management decisions for the same field. Although the multiplicative P‐index was designed to address the limitation of additive P‐index, the multiplicative P‐index from Tennessee exhibited substantial disparities in P loss risk categorization compared to observed P losses and is not directly applicable to Alabama farms. Moreover, the discrepancy of P‐index between Alabama and Tennessee P‐indices was reported by Osmond et al. (2012, 2006), where a field with medium P loss risk for Alabama was categorized as very high risk for Tennessee P‐index.

The component P‐index from Georgia demonstrated a better correlation (*r* = 0.42) between DRP loads and P‐index score than multiplicative (Figure 3c); however, it was not significant. Similarly, the relationship between TPP and TP loads with P‐index score was not significant, and some fields with minimal P loads were incorrectly classified as high‐risk. Compared to additive and multiplicative, the component P‐index showed better trend directionally (higher P‐index score for higher P loading sites). Osmond et al. (2017, 2012) reported that component‐based P‐index from Georgia better predict the dissolved P (*r* = 0.85) and TP (*r* = 0.72) in runoff. This indicated that the component‐based P‐index has a better potential to predict dissolved P in runoff. Further research is needed to evaluate the applicability of a component‐based P‐index for Alabama farms and to guide its restructuring for more accurate and site‐relevant P loss assessment.

Previous studies on the evaluation of the performance of P‐index from different states using P‐loss data showed a good correlation. For instance, the Ohio P‐index (Williams et al., 2016) and the Minnesota (Reitmeier et al., 2024) and Wisconsin (Good et al., 2012) P‐indices were directionally correct. These P‐index have been revised over time and are structured as component‐based P‐indices, and these states are continuing efforts to refine these indices. These findings suggest an urgent need for structural improvements to the Alabama P‐index, including transitioning to a component‐based framework, to better align with P loss dynamics to improve water quality outcomes.

### 3.4 Proposed modification to Alabama P‐index

**FIGURE 4:**
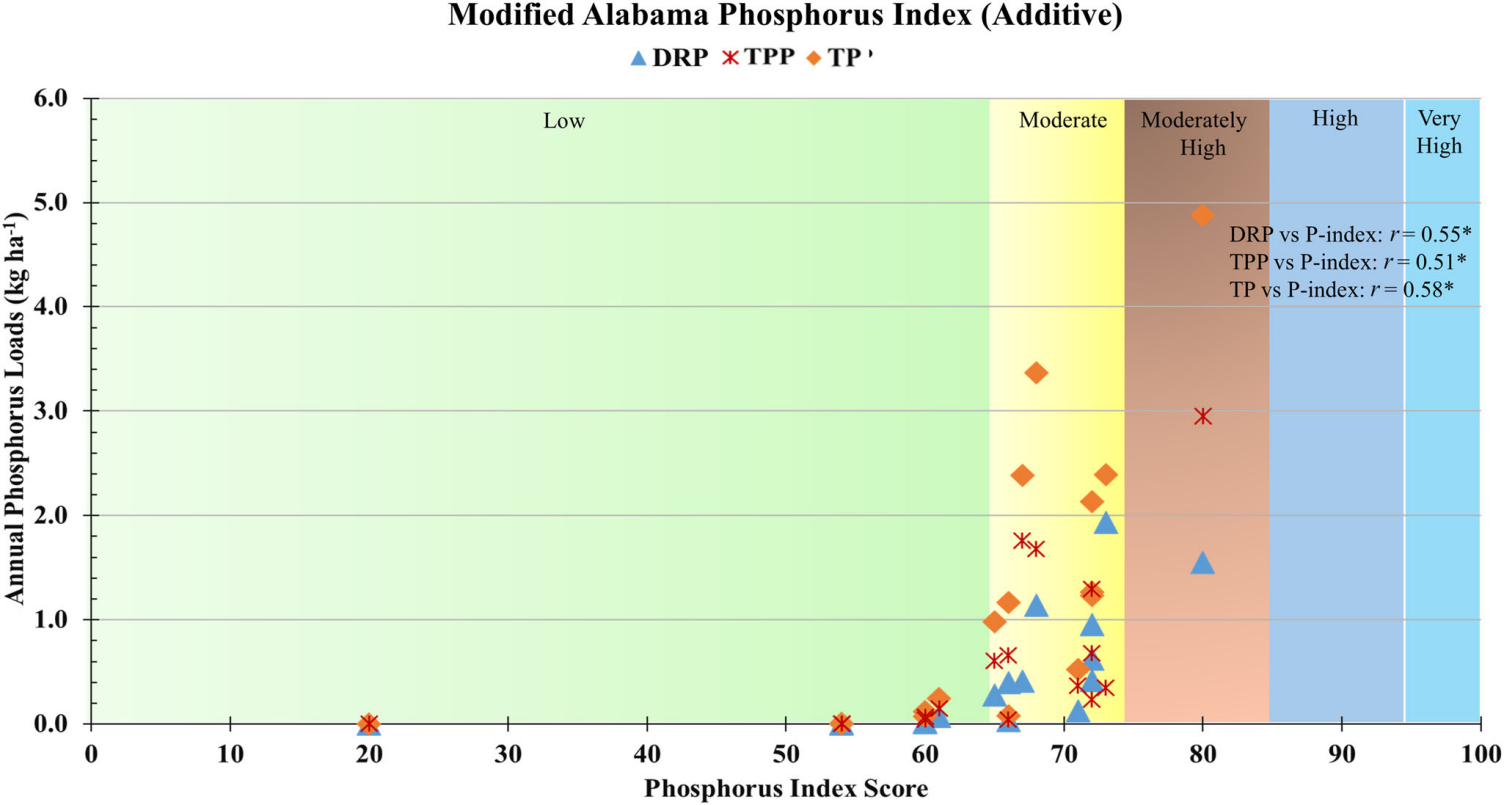
Relationship between annual phosphorus loads [dissolved reactive phosphorus (DRP), total particulate phosphorus (TPP), and total phosphorus (TP)] with phosphorus index score for modified Alabama phosphorus index (additive). The phosphorus index (P‐index) score <65 is low, 66–75 is moderate, 75–85 is moderately high, 86–95 is high, and >95 is very high‐risk category as differentiated by different colors; * represents statistically significant relationship.

As demonstrated in this study, the additive Alabama P‐index ratings underestimate potential P losses from fields. Until Alabama can develop its own component index, we modified the Alabama index (Table 4). The modified Alabama P‐index demonstrated improved correlation coefficients with DRP (*r* = 0.55), TPP (*r* = 0.51), and TP (*r* = 0.58) loads, and were statistically significant (*p* < 0.05) (Figure 4). The modified P‐index demonstrated improved directional accuracy in predicting P loss, reflecting enhanced predictive capability and greater alignment of P‐index scores with observed P loss data. These results suggest that the modified P‐index can serve as a reliable interim tool for P loss risk assessment for Alabama.

**TABLE 4:** Modified version of Alabama phosphorus index.

S.N.	Source characteristics		Weighting	Value rating				
				Low				Very high
				0 Point	1 Point	2 Points	4 Points	8 Points
**1**	P saturation ratio (M3) (0–6 inch)		**3**		<0.05	0.05–0.09	0.1–0.15	>0.15
**2**	P application rate (lbs/P_2_O_5_/ac/year)	Traditional application	**2**	**…**.	<60	60–120	121–180	>180
		Precision application	**1**	**…**.	<60	60–120	121–180	>180
**3**	P application method		3	….	Injected deeper than 2 "	Incorporated within 3 days, sprinkler applied or surface applied treated	Surface applied and incorporated within 4 to 30 days	Surface applied, not incorporated
**4**	P application time		**2**		Jun–Aug	Sept–Oct	Mar–May	Nov–Feb
**5**	Grazing animals		1	None	No access to water and not fed in sensitive area	Restricted access to water and not fed in sensitive area	Unlimited access to water and/or fed in sensitive area <100 animals	Unlimited access to water and/or fed in sensitive area >100 animals
Transport characteristic								
**6**	Erosion rate (t)		3	<3	3–5	5–10	10–15	>15
**7**	Hydrologic soil group	Common soil health	3	**…**.	A	B	C	D
		Improved soil health	2	**…**.	A	B	C	D
**8**	Field slope		**2**	<1%	1%–3%	3%–5%	5%–8%	>8%
**9**	P application distance to water (ft)		**2**	>400	201–400	101–200	50–100	<50
**10**	Vegetative buffer width (ft)		2	≥50 or not required	30–49	20–29	10–19 t	<10
**11**	Impaired outstanding or critical habitat waters (ft)		3	Not in watershed	>400	201–400	101–200	<100

### 3.5 Recommendations for P‐index improvements and research directions

Decades of research and refinement have significantly advanced the understanding of P management through the development of P‐indices. The transition from additive models to more sophisticated multiplicative and component‐based approaches has become increasingly necessary. Studies by Gburek et al. (2000) and Bolster et al. (2012) have demonstrated that shifting from additive to multiplicative or component‐based structures improves the performance and accuracy of P‐indices by better capturing the interactions between source and transport factors. To enhance the accuracy of P loss assessments, it is essential to evaluate individual factors within both source and transport components. This includes assigning specific coefficients for different types of manure and fertilizers—a methodology successfully implemented in the Pennsylvania P‐index (Weld et al., 2007) and the Georgia P‐Index (Cabrera et al., 2012). Such detailed parameterization allows for more precise risk categorization and better reflects the variability in P sources and transport mechanisms across diverse agricultural systems. Another critical aspect of P‐index improvement involves estimating erosion rates using RUSLE2. Although RUSLE2 is widely used, it has been reported to have larger uncertainty in predicting erosion, necessitating the development of adjustment factors to better capture soil erosion (Good et al., 2012; Osmond et al., 2012; Reitmeier et al., 2024).

The dataset used in this study represents row crop fields monitored over a 3‐year period, providing a foundational assessment of the Alabama P‐index. However, future improvements will require long‐term edge‐of‐field monitoring data across a broader range of management systems, including pasturelands and diverse soil types. Long‐term datasets are essential for capturing the variability in P loss dynamics and for calibrating and validating the index under varying environmental and agronomic conditions.

Moreover, periodic revisions are necessary to ensure that the index reflects the latest scientific advancements and P‐based management practices (Reitmeier et al., 2024). Given the inherent uncertainties in field‐based measurements, continued research is critical to address limitations and enhance the performance of the P‐index. Engaging farmers, policymakers, and other stakeholders in the refinement process will help ensure that the tool is both scientifically robust and practically applicable. Drawing on successful approaches from other states, the Alabama P‐index has significant potential for improvement and can evolve into a more reliable and widely adopted P‐risk assessment tool.

## 4 CONCLUSIONS

The effectiveness and reliability of the P‐index in assessing P loss risk from agricultural fields depend on several key factors, including its structural framework (additive, multiplicative, or component‐based) and the inclusion of relevant source and transport parameters. This study evaluated the performance of the Alabama P‐index using edge‐of‐field monitoring data from Alabama farms. To explore alternative approaches, P loss risk predictions were also assessed using multiplicative (Tennessee) and component‐based (Georgia) P‐indices applied to the same sites.

The misclassification of sites with relatively high P loads into low‐risk categories revealed limitations in the Alabama P‐index's ability to accurately assess P loss risk. Similar discrepancies were observed with the multiplicative formulation, indicating that structural changes alone may not resolve performance issues without appropriate parameterization. However, when the Alabama index was modified to incorporate the P saturation ratio, timing of P application, and adjusted weighting factors, its performance improved significantly, demonstrating better alignment with measured P loads. While the component‐based model from Georgia did not outperform the modified Alabama index when directly applied, it shows promise and warrants further restructuring to suit Alabama's specific conditions. Future research should prioritize transitioning the Alabama P‐index from its current additive structure to a component‐based framework while using the modified version as an interim tool for P loss risk assessment.

## AUTHOR CONTRIBUTIONS

**Anjan Bhatta**: Conceptualization; data curation; formal analysis; methodology; resources; software; validation; visualization; writing—original draft. **Rishi Prasad**: Conceptualization; funding acquisition; investigation; project administration; resources; software; supervision; validation; writing—review and editing. **Debolina Chakraborty**: Conceptualization; formal analysis; investigation; project administration; software; supervision; writing—review and editing. **Dexter B. Watts**: Writing—review and editing. **Henry A. Torbert**: Writing—review and editing. **Peter Kleinman**: Writing—review and editing.

## CONFLICT OF INTEREST STATEMENT

The authors declare no conflicts of interest.

## Supporting information




**Supplementary Table 1**: Field characteristics and management practices across six Alabama watersheds during the study period.
**Supplementary Table 2**: Number of Site‐Years Classified by USDA‐NRCS Nutrient Management Risk Categories and Corresponding Standardized Phosphorus Loading Ranges *(Based on USDA‐NRCS Title 190 – National Instruction, Part 302: Nutrient Management Policy Implementation, 2011)*
**Supplementary Figure 1**: The map and watershed boundaries showing grid sampling points within the watersheds.
**Supplementary Figure 2**: Spatial Patterns of Soil Test Phosphorus (Mehlich‐1, 0–15 cm) in Alabama Watersheds: North (W1, W2), Central (W5, W6), and South (W3, W4) from 2021 to 2023.
**Supplementary Figure 3**: PSR_M3_ distribution across three years (2021, 2022 and 2023) for 0 – 15 cm depths for three watersheds studied.
**Supplementary Figure 4**: Phosphorus Index Scores and measured phosphorus loads (DRP, TPP, TP) across North, South, and Central Alabama sites.
