# Supplementary material for: Evaluation of Alabama phosphorus index using edge‐of‐field monitoring data
Source: J Environ Qual. 2026 Feb 13;55(1):e70152. doi: 10.1002/jeq2.70152 (PMC12905517; doi:10.1002/jeq2.70152)
Supplement: Supplementary file 1 — Supplementary Table 1: Field characteristics and management practices across six Alabama watersheds during the study period. Supplementary Table 2: Number of Site‐Years Classified by USDA‐NRCS Nutrient Management Risk Categories and Corresponding Standardized Phosphorus Loading Ranges (Based on USDA‐NRCS Title 190 – National Instruction, Part 302: Nutrient Management Policy Implementation, 2011) Supplementary Figure 1: The map and watershed boundaries showing grid sampling points within the watersheds. Supplementary Figure 2: Spatial Patterns of Soil Test Phosphorus (Mehlich‐1, 0–15 cm) in Alabama Watersheds: North (W1, W2), Central (W5, W6), and South (W3, W4) from 2021 to 2023. Supplementary Figure 3: PSRM3 distribution across three years (2021, 2022 and 2023) for 0 – 15 cm depths for three watersheds studied. Supplementary Figure 4: Phosphorus Index Scores and measured phosphorus loads (DRP, TPP, TP) across North, South, and Central Alabama sites. [file JEQ2-55-0-s001.docx]

**Evaluation of Alabama phosphorus index using edge-of-field monitoring data**

Anjan Bhatta^1^ Rishi Prasad^1*^ Debolina Chakraborty^2^ Dexter B. Watts^3^ Henry A. Torbert^3 4^Peter Kleinman

^1^Department of Crop, Soil, and Environmental Sciences, Auburn University, Auburn, Alabama

^2^Department of Biosystems Engineering, Auburn University, Auburn, Alabama

^3^USDA-ARS, National Soil Dynamics Lab, Auburn, Alabama

^4^USDA-ARS-Soil Management and Sugarbeet Research Unit, Fort Collins, Colorado

**Supplementary Table 1**: Field characteristics and management practices across six Alabama watersheds during the study period.

| Characteristics /Practices | Watershed 1 | Watershed 2 | Watershed 3 | Watershed 4 | Watershed 5 | Watershed 6 |
| --- | --- | --- | --- | --- | --- | --- |
| Watershed area (ha) | 10.52 | 37.23 | 2.10 | 1.87 | 2.31 | 3.52 |
| Site | North Alabama | North Alabama | South Alabama | South Alabama | Central Alabama | Central Alabama |
| Dominant soil series | Decatur silty clay loam, eroded  Abernathy-Emory silt loams | Decatur silt loam  Decatur silty clay, severely eroded | Eunola sandy loam | Eunola sandy loam | Marlboro loamy sand | Marlboro loamy sand |
| Cropping system | | | | | | |
| 2021/22 | Soybean (Jul – Oct) Fallow (Nov – Mar) | Soybean (Jul – Oct) Fallow (Nov – Mar) | Peanuts (May – Oct) – Cover crop (Nov – Mar) | Peanuts (May – Oct) – Cover crop (Nov – Mar) | Peanuts (May - Oct) – Cover crop (Nov - Apr) | Peanuts (May - Oct) – Cover crop (Nov - Apr) |
| 2022/23 | Corn (Apr - Aug) – Winter wheat (Nov - May) | Corn (Apr - Aug) – Winter wheat (Nov - May) | Corn (Mar - Jul) – Soybean (Jul – Nov) – Cover crop (Nov – Mar) | Corn (Mar - Jul) – Soybean (Jul – Nov) – Cover crop (Nov – Mar) | Cotton (May - Oct) – Cover crop (Nov – Mar) | Cotton (May - Oct) – Cover crop (Nov – Mar) |
| 2023/24 | Soybean (May – Oct) – winter wheat (Nov - May) | Soybean (May – Oct) – winter wheat (Nov - May) | Cotton (May – Oct) – Cover crop (Nov – Mar) | Cotton (May – Oct) – Cover crop (Nov – Mar | Corn (May – Oct) – Cover crop (Nov – Mar) | Corn (May – Oct) – Cover crop (Nov – Mar) |
| Rate and time of manure and fertilizer application | | | | | | |
| 2021 | 4.5 Mg ha^-1^ poultry litter (Jun) | 4.5 Mg ha^-1^ poultry litter (Jun) | 0-140-280 kg ha^-1^ of N-P_2_O_5_-K_2_O for Peanuts | 0-140-280 kg ha^-1^ of N-P_2_O_5_-K_2_O for Peanuts | No application | No application |
| 2022 | 9 Mg ha^-1^ poultry litter (Mar. and Sep.) | 9 Mg ha^-1^ poultry litter (Mar. and Sep.) | 170: 100:170 and 45:100:280 kg ha^-1^ of N-P_2_O_5_-K_2_O for corn and soybean, respectively | 170: 100:170 and 45:100:280 kg ha^-1^ of N-P_2_O_5_-K_2_O for corn and soybean, respectively | 67 kg ha-1 of P_2_O_5_ (May) | 67 kg ha^-1^ of P_2_O_5_ (May) |
| 2023 | 4.5 Mg ha^-1^ poultry litter (May) | 4.5 Mg ha^-1^ poultry litter (May) | 140:100:140 kg ha^-1^ of N-P_2_O_5_-K_2_O for cotton | 140:100:140 kg ha^-1^ of N-P_2_O_5_-K_2_O for cotton | 4.5 Mg ha^-1^poultry litter (Mar) | 4.5 Mg ha^-1^poultry litter (Mar) |
| Note: Information on dominant soil series were derived from USDA Web Soil Survey. (accessed on September 10, 2024). | | | | | | |

**Supplementary Table 2**: Number of Site-Years Classified by USDA-NRCS Nutrient Management Risk Categories and Corresponding Standardized Phosphorus Loading Ranges *(Based on USDA-NRCS Title 190 – National Instruction, Part 302: Nutrient Management Policy Implementation, 2011)*

| **Risk category** | **Phosphorus loadings range (kg ha^-1^ yr^-1^)** | **Number of site years** | | |
| --- | --- | --- | --- | --- |
|  |  | **Dissolved reactive P (DRP)** | **Total particulate P (TPP)** | **Total P (TP)** |
| Low | < 2.2 | 17 | 16 | 13 |
| Medium | 2.2 – 5.6 | - | 1 | 4 |
| High | >5.6 | - | - | - |

**Supplementary Figures:
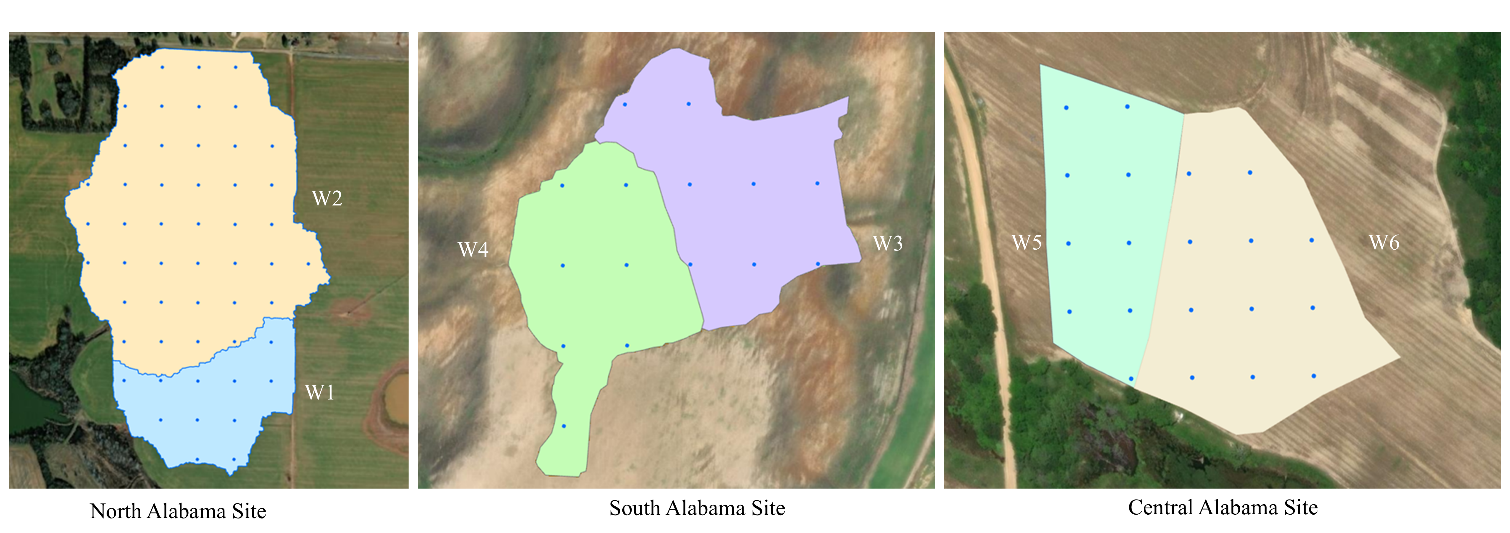
**

**Supplementary Figure 1**: The map and watershed boundaries showing grid sampling points within the watersheds.


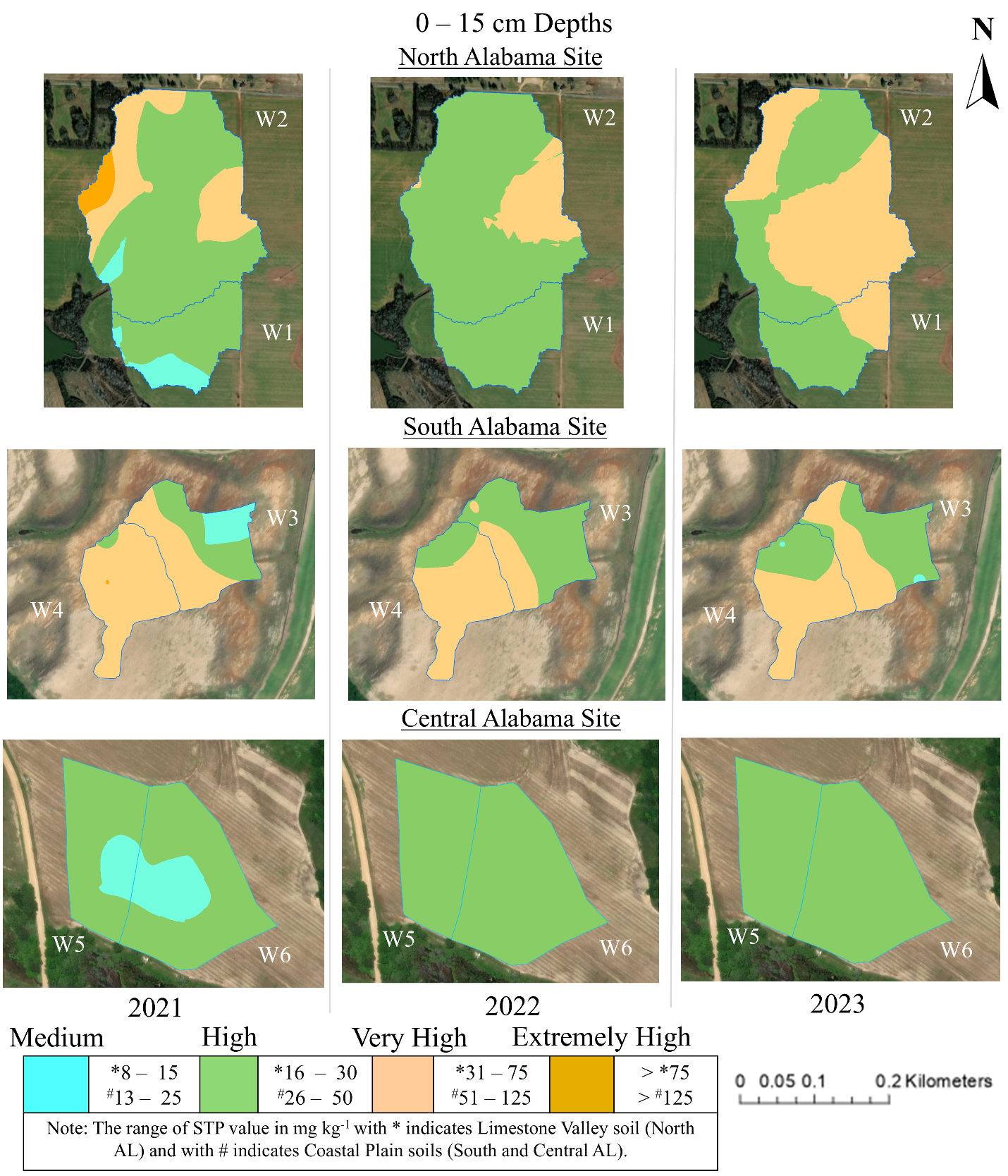


**Supplementary Figure 2**: Spatial Patterns of Soil Test Phosphorus (Mehlich-1, 0–15 cm) in Alabama Watersheds: North (W1, W2), Central (W5, W6), and South (W3, W4) from 2021 to 2023

**
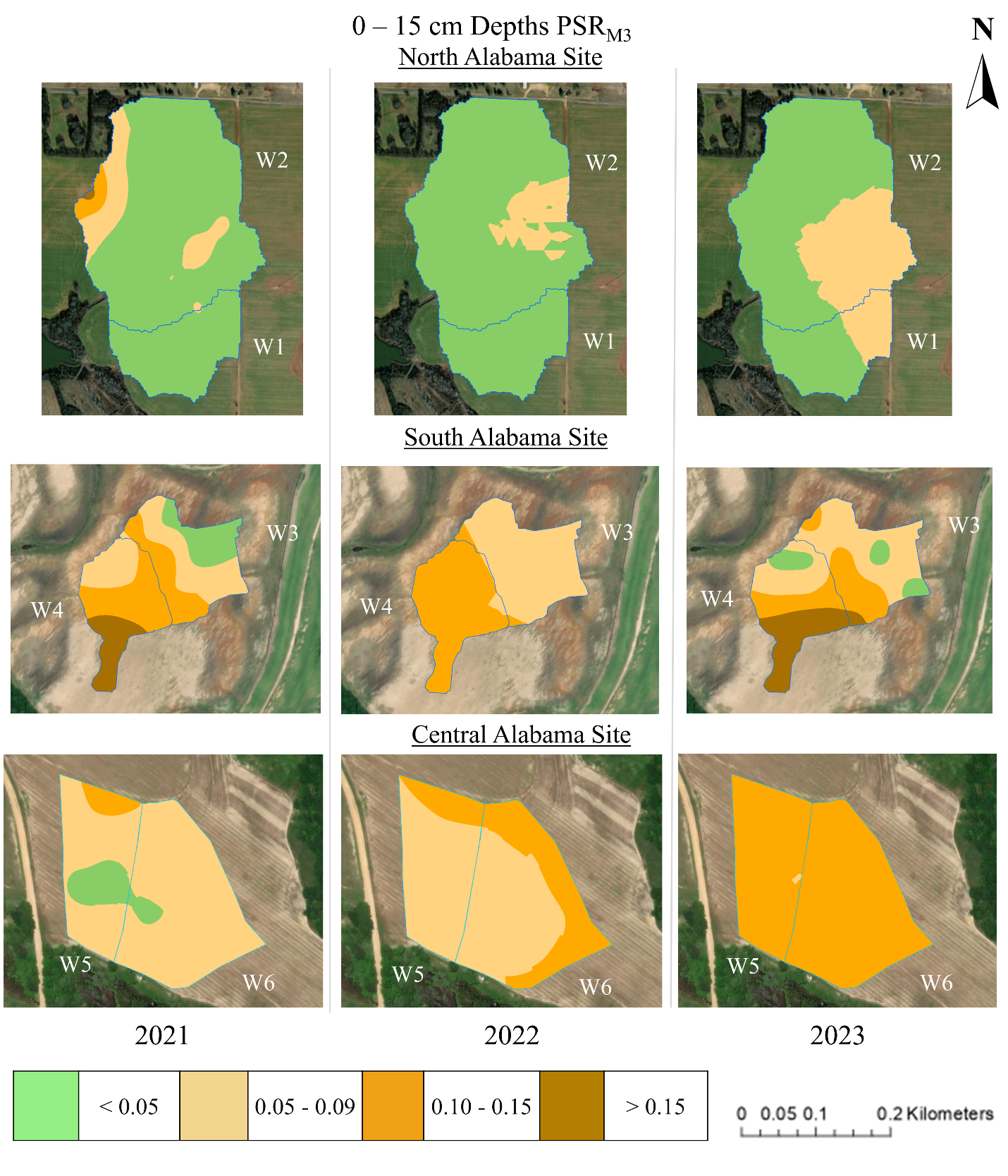
**

**Supplementary Figure 3**: PSR_M3_ distribution across three years (2021, 2022 and 2023) for 0 – 15 cm depths for three watersheds studied.


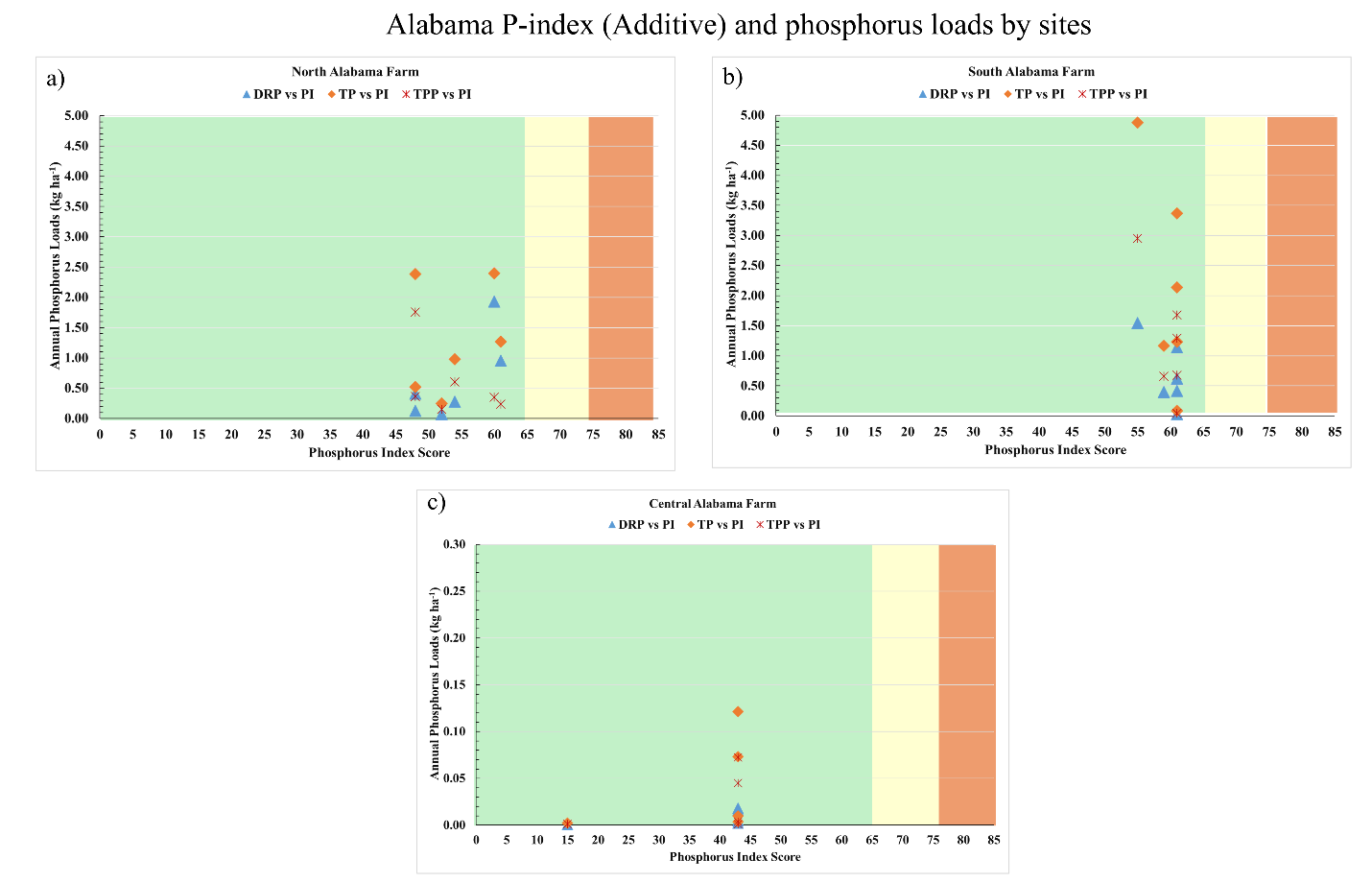


**Supplementary Figure 4**: Phosphorus Index Scores and measured phosphorus loads (DRP, TPP, TP) across North, South, and Central Alabama sites
